# Population genomics reveals how 5 ka of human occupancy led the Lima leaf-toed gecko (*Phyllodactylus sentosus*) to the brink of extinction

**DOI:** 10.1038/s41598-023-45715-x

**Published:** 2023-10-27

**Authors:** Alejandra Arana, Juan Esteves, Rina Ramírez, Pedro M. Galetti, José Pérez Z., Jorge L. Ramirez

**Affiliations:** 1 Facultad de Ciencias Biológicas, Universidad Nacional Mayor de San Marcos, Lima, Peru; 2 Departamento de Genética e Evolução, Universidade Federal de São Carlos, São Carlos, SP 13565-905 Brazil

**Keywords:** Population genetics, Herpetology

## Abstract

Small species with high home fidelity, high ecological specialization or low vagility are particularly prone to suffer from habitat modification and fragmentation. The Lima leaf-toed gecko (*Phyllodactylus sentosus)* is a critically endangered Peruvian species that shelters mostly in pre-Incan archeological areas called *huacas*, where the original environmental conditions are maintained*.* We used genotyping by sequencing to understand the population genomic history of *P. sentosus*. We found low genetic diversity (He 0.0406–0.134 and nucleotide diversity 0.0812–0.145) and deviations of the observed heterozygosity relative to the expected heterozygosity in some populations (F_is_ − 0.0202 to 0.0187). In all analyses, a clear population structuring was observed that cannot be explained by isolation by distance alone. Also, low levels of historical gene flow were observed between most populations, which decreased as shown in contemporary migration rate analysis. Demographic inference suggests these populations experienced bottleneck events during the last 5 ka. These results indicate that habitat modification since pre-Incan civilizations severely affected these populations, which currently face even more drastic urbanization threats. Finally, our predictions show that this species could become extinct in a decade without further intervention, which calls for urgent conservation actions being undertaken.

## Introduction

Habitat loss, degradation and fragmentation stand out among the main anthropogenic threats to biodiversity^1,2^. One of the primary consequences of these anthropogenic actions is the reduced connectivity of patches of natural habitat, which leads to decreased gene flow among isolated populations that can result in depleting their genetic diversity and increasing genetic drift^3–5^.

Reptiles are a highly diverse taxonomic group that, despite its ecological importance^6^ and high sensitivity to human landscape modification^7,8^, has been often overlooked in studies on the effect of anthropogenic habitat alterations^9^. Although this is a growing field, there is currently a bias toward studies done on temperate countries and on mammals and birds^9^. Considering the recent assessment that 21% of reptile species are endangered^10^ and the current expansion of cities and human settlements, the scant studies of the effects of human landscape change on reptile population genetics, especially in the Neotropics, represent a knowledge gap that needs to be addressed in order to develop effective conservation strategies^9^.

**Figure 1:**
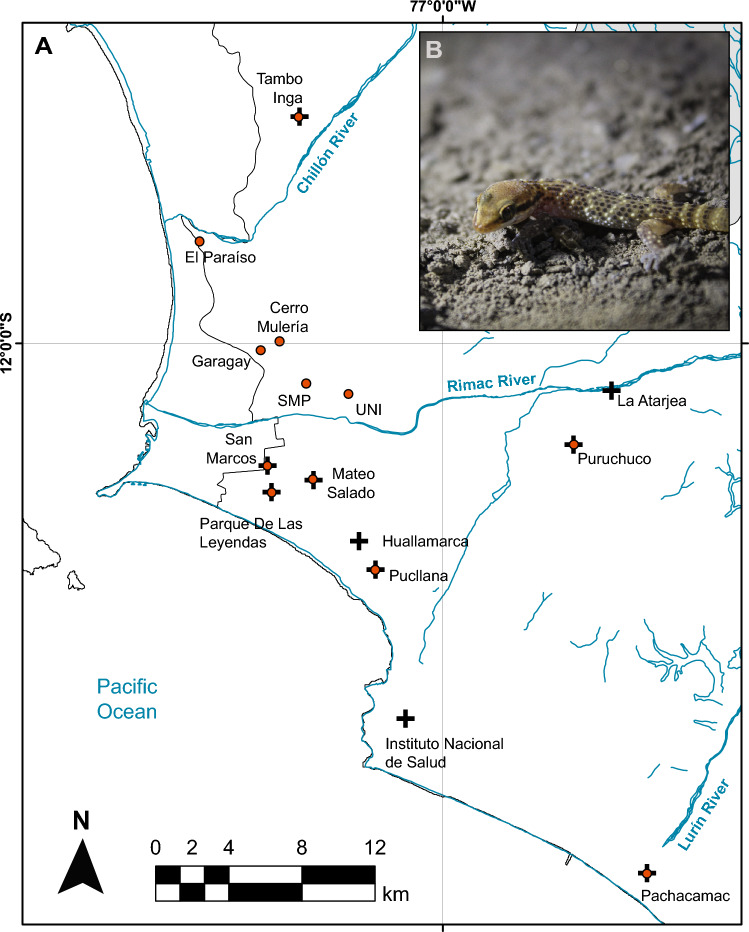
Distribution map of the Lima leaf-toed gecko. (**A**) Sites where the species had been previously recorded^11,13–15^ in the province of Lima (cross) and sampling localities used in this study (circle). (**B**) Photograph of a juvenile Lima leaf-toed gecko found in the Pachacamac *huaca*. Photo by A. Arana. Map created in ArcGIS 10.3. Free vector data from https://www.geogpsperu.com/2014/03/base-de-datos-peru-shapefile-shp-minam.html.

Small species with high home fidelity, high ecological specialization or low vagility are particularly prone to experience adverse effects due to habitat fragmentation^5^. As a case in point, the Lima leaf-toed gecko (*Phyllodactylus sentosus),* endemic of the city of Lima (Peru) and classified as critically endangered in the IUCN Red List of Threatened Species^11^, is constrained to small patches mainly represented by pre-Incan archeological areas called *huacas* and hilltops^12–15^ (Fig. 1), which feature arid soil with scant vegetation and small rocks that resemble the natural habitat previous to human occupancy^11^. Nowadays, these areas are isolated from each other by completely urbanized areas in Lima, being estimated not to exceed the 500 adult individuals in *huacas* with the largest census population size^16–18^. Also, unlike other *Phyllodactylus* species, *P. sentosus* has low climbing abilities due to their toe structure, which reduces the probability of dispersion and escape from invasive local threats^15^.

*Huacas* have been studied as islands of arid habitat in both urban and agricultural landscapes along the coast of Peru, affecting species distributions^19,20^. However, the effect of this “archeological landscape” on wildlife population genetics has not been analyzed yet. In recent years, the study of the effect of urbanization and human settlements on connectivity and gene flow between populations of wild species has been enhanced by the development of reduced representation sequencing techniques, such as genotyping by sequencing (GBS), which allows the discovery of thousands of genome-wide single nucleotide polymorphisms (SNPs) without the need for a reference genome^21–24^. These techniques have also increased the opportunities to explore the demographic history of populations and to perform population viability analysis to inform conservation actions.

In this study, we used GBS data to estimate genetic diversity and assess inbreeding to evaluate the current genetic status of Lima leaf-toed gecko populations. Also, we evaluated the genetic structure, historical and contemporary gene flow and demographic history to gain insights into the population genetics of the species. Finally, we performed a Population Viability Analysis (PVA) to simulate future scenarios of this species in absence of conservation actions.

## Results

### Data generation

We generated two datasets of GBS data for a total of 50 individuals from 12 localities of the Lima leaf-toed gecko. In the first dataset (i), 147 856 SNPs were obtained using 0.85 as the clustering threshold and 33 as the minimum number of samples that must have information at a locus for it to be retained. From the second dataset (ii), which had no missing data, 10,869 SNPs were obtained.

### Genetic diversity and inbreeding

**Figure 2:**
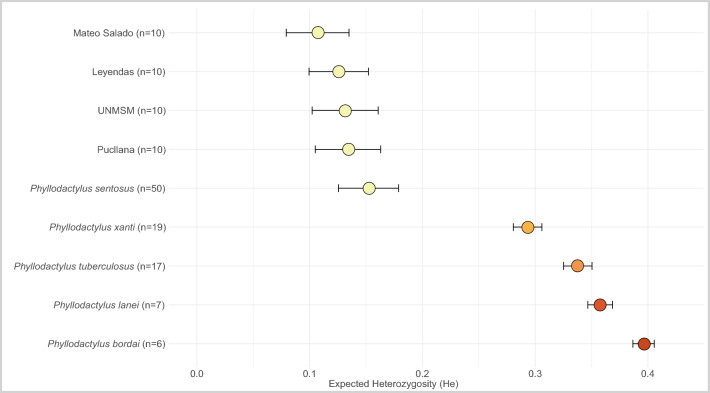
Comparison of expected heterozygosity among *Phyllodactylus* species. The four largest populations as well as the global value of genetic diversity for *P. sentosus* compared to four other species belonging to the same genus.

Population-level genetic diversity estimates are given in Table 1. The values observed were quite low for all populations (see “Discussion”). Expected Heterozygosity (He) values ranged from 0.0406 to 0.134 in *P. sentosus* populations, while values for other four *Phyllodactylus* species analyzed ranged from 0.2933 to 0.3961 (Fig. 2).

**Table 1:** Genetic diversity indexes (He, π) obtained for each population.

Population	n	Expected Heterozygosity (He)	Nucleotide diversity (π)	F_IS_
Pucllana	10	0.134	0.1427	− 0.0202 (σ = 0.0135)
San Marcos	10	0.1315	0.1392	− 0.0031 (σ = 0.0115)
Parque de las Leyendas	10	0.1258	0.1331	0.0187 (σ = 0.0118)
Mateo Salado	10	0.1071	0.1139	− 0.0058 (σ = 0.0115)
Cerro Mulería	2	0.1066	0.145	–
SMP	2	0.0915	0.123	–
El Paraíso	1	0.0513	0.1025	–
UNI	1	0.0519	0.1039	–
Pachacamac	1	0.0548	0.1096	–
Puruchuco	1	0.0521	0.1041	–
Tambo Inga	1	0.0406	0.0812	–
Garagay	1	0.0408	0.0816	–

F_IS_ values were not calculated for some populations due to their small sample size.

The results of Nucleotide Diversity (π) ranged between 0.0812 and 0.145. The population of Cerro Mulería has the highest value of Nucleotide Diversity (π) despite the fact that only 2 individuals were collected in this population. The deviation of the observed heterozygosity relative to the expected heterozygosity (F_IS_) ranged between –0.0202 and 0.0187 (Table 1).

### Population genetic structure

**Figure 3:**
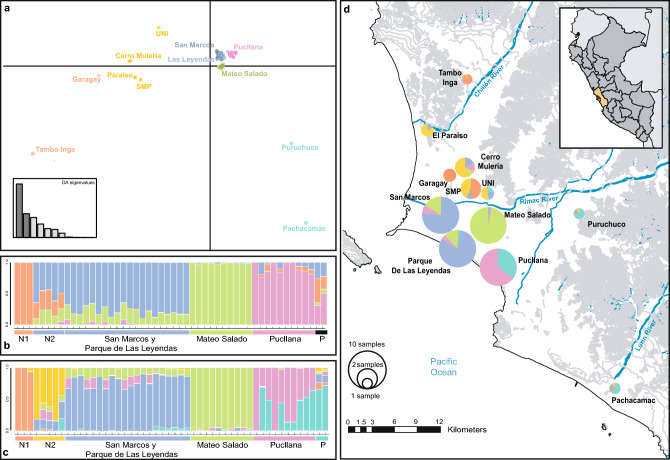
Genetic population structure of *Phyllodactylus sentosus* in Lima. (**a**) Discriminant analysis of principal components (DAPC), the axes represent the first two discriminant components. (**b**) STRUCTURE results from 3737 SNPs, with K = 4. (**c**) STRUCTURE results from 15,816 SNPs, with K = 6. Group P includes the Puruchuco and Pachacamac samples. Group N2 corresponds to samples from Paraíso, Mulería, UNI and one from SMP. Group N1 includes samples from Tambo Inga, Garagay and one from SMP (San Martin de Porres). (**d**) Map of the evaluated localities and the average of the coefficients of individual membership to genetic groups in each population from the STRUCTURE analysis with 15,816 SNPs. The size of the pie charts indicates the sample size. Altitudinal relief is represented in gray lines (altitudes higher than 25 m). Map created in ArcGIS 10.3. Free vector data from https://www.geogpsperu.com/2014/03/base-de-datos-peru-shapefile-shp-minam.html.

In all analyses, a clear population structuring was observed, except Huaca San Marcos and Parque de Las Leyendas that were grouped in a single genetic cluster in most analyses (Fig. 3, see Supplementary Figs. S1, S2 online), corresponding to the pre-Hispanic Maranga archeological complex (see “Discussion”).

There are two main rivers that run through the city of Lima: Rímac River and Chillón River (Fig. 3d). In the DAPC analysis, the horizontal axis separates the Tambo Inga population (the only one north Chillón River) from the rest of populations (Fig. 3a). Also, the locations north Rímac River (Cerro Mulería, Garagay, Paraíso, Tambo Inga, San Martín de Porres, and UNI) were separated from the south Rímac River (Mateo Salado, Pachacamac, Parque de Las Leyendas, Pucllana, Puruchuco, and San Marcos) (Fig. 3a).

STRUCTURE analyses revealed high levels of population structuring (Fig. 3d). The dataset which included only SNPs (3373) called in the entire sample set, returned K = 4 as the most likely number of genetic clusters (Fig. 3b). Then we analyzed a larger SNP dataset (15,816) called in 90% of samples in each population, with K = 6 as the most likely number of genetic clusters (Fig. 3c). The main difference between the results of these two analyses occurred in the populations north Rímac River, which showed population structuring (Fig. 3b,c). At K = 4, only one genetic group emerged, while at K = 6 the populations north of the river split into two genetic clusters, called herein as N1 and N2, with the former being composed of populations near the river. In Mateo Salado population, the coefficients of individual genetic group membership (q) obtained from the samples were particularly high (> 0.97).

### Gene flow

**Figure 4:**
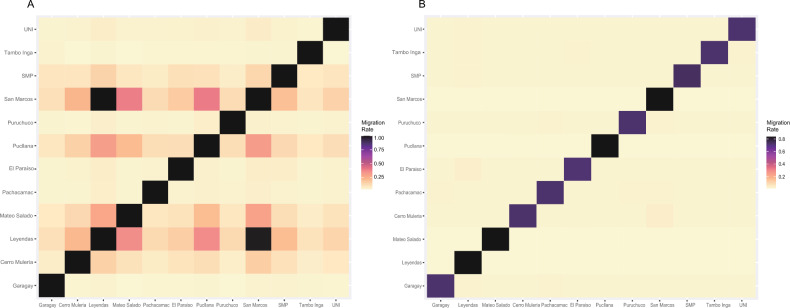
Gene flow among Lima leaf-toed gecko populations. Estimates of historical gene flow (**A**) and contemporary gene flow (**B**). Migration rate* values shown in (**A**) are GST values as calculated by divMigrate^25^. Migration rate^**+**^ values shown in (**B**) are proportion of migrants as calculated by BA3-SNPs^26,27^.

The divMigrate analysis on the dataset with no missing data yielded values of relative migration rates ranging from 0.03817 (between Mateo Salado and Tambo Inga) to 1 (between San Marcos and Parque de Las Leyendas) (Fig. 4A). The populations at the extremes of the geographical distribution of this species show the lowest migration rates, while those among the four largest census population size^16–18^ (San Marcos, Parque de Las Leyendas, Pucllana and Mateo Salado: Fig. 4, see Supplementary Table S3 online) were slightly higher, but still lower than 0.4, with exception of the populations of San Marcos and Parque de Las Leyendas that showed a high bidirectional gene flow with migration values of 0.99 and 1 (Fig. 4).The results also indicate especially low values of migration to and from Tambo Inga, the only locality north Chillón River (see Supplementary Table S3 online). On average, Tambo Inga had the lowest migration values in both directions, followed by populations located north Rímac River, such as Garagay and El Paraíso, and populations at the southern (Pachacamac) and eastern (Puruchuco), at the extremes of the species distribution (see Supplementary Table S3 online). The Mantel test showed a non-significant positive correlation (r = 0.30; p-value = 0.08), suggesting that gene flow patterns cannot be explained solely by isolation by distance.

On the other hand, contemporary gene flow rates are low across all localities, with the proportion of migrants in most populations being lower than 0.07 (< 7%). Contemporary migration rates between localities range from 0.0149 to 0.166, the latter being the proportion of migrants from Parque de Las Leyendas present in San Marcos. Conversely, the proportion of migrants from San Marcos in Parque de Las Leyendas is 0.0604, showing a lower migration rate than the ones observed between SMP and Parque de Las Leyendas (0.071), and Muleria and San Marcos (0.0712).

### Demographic history

**Figure 5:**
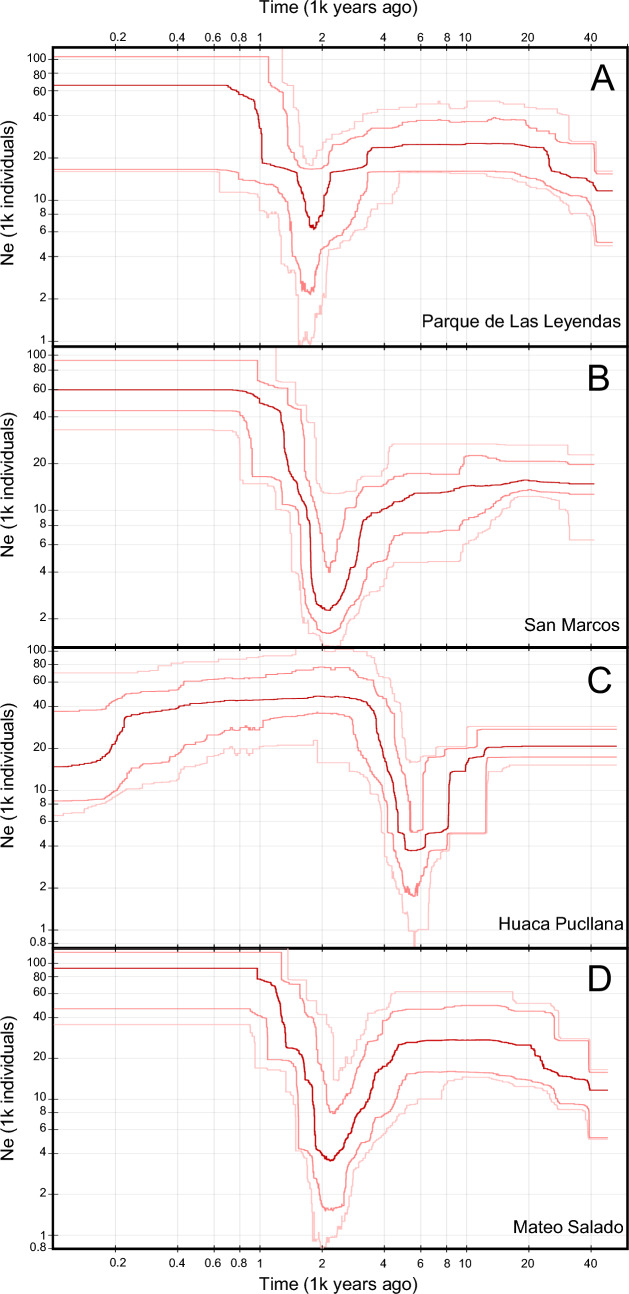
Demographic history of *P. sentosus*. Stairway plot demographic inference of the populations of Parque de Las Leyendas (**A**), San Marcos (**B**), Mateo Salado (**C**), and Huaca Pucllana (**D**). The red line shows the median estimate of the effective population size (Ne). The pink lines indicate the 75% (dark) and 95% (light) confidence intervals.

The demographic history estimation results for Parque de Las Leyendas, San Marcos, Mateo Salado and Huaca Pucllana revealed the same pattern in all populations, with an accentuated bottleneck and a posterior increase in population size (Fig. 5). The effective population size is estimated to have decreased 3100–5000 years ago and reached its minimum about 2000 years ago in Parque de Las Leyendas, San Marcos, and Mateo Salado. On the other hand, at Pucllana we estimated that the effective population began to decrease more than 10 thousand years ago, and dropped drastically 8000 years ago, reaching its minimum about 5000 years ago (Fig. 5C). The population size started to increase after the bottleneck and remained stable, except for Pucllana, where a decline in the effective population was observed about 200 years ago (Fig. 5C).

### Population viability analysis

PVA using Vortex showed that all populations would experience a decrease in their population size resulting in extinction (see Supplementary Fig. S5 online). All simulations resulted in mean extinction times of 9.7 years or less (Table 2).

**Table 2:** Results of Vortex simulations for the five populations after 30 years.

N0	stoch-r	PE	N-all	GeneDiv	MeanTE (years)
50	− 0.182	1	0	0	8.2
100	− 0.1724	1	0	0	8.8
200	− 0.1635	0.998	0.01	0.0757	9.4
500	− 0.1545	0.989	0.06	0.1008	9.7
1000	− 0.1473	0.963	0.3	0.1189	9.2

N0: initial census population size; stoch-r: mean rate of stochastic growth or decline of the population; PE: Probability of extinction; N-all: mean population size at the end of the simulation; GeneDiv: genetic diversity, MeanTE: mean extinction time.

## Discussion

Here we report the first population genomics survey of the critically endangered Lima leaf-toed gecko *P. sentosus*. According to our results, the populations of this species present low levels of genetic diversity, highly structured populations, and negligible levels of contemporary gene flow between populations. All these results together with the PVA simulations showed an unfavorable scenario for the species.

The locations that host the largest populations of this species are San Marcos, Parque de Las Leyendas, Pucllana and Mateo Salado^16–18^. Large population size is expected to maintain higher levels of genetic diversity. However, even in these localities, very low values relatives of gene diversity were found (He < 0.135). Our analysis of He values for four other *Phyllodactylus* species revealed values around twice as high as those obtained for *P. sentosus* when considering all sampled individuals. This reduced genetic diversity could be explained by a recent bottleneck (see below) and by the fragmentation of small populations in restricted areas with limited resources^28^.

Inbreeding in wildlife populations increases homozygosis, reduces genetic diversity, and can represent an important genetic threat if it derives into inbreeding depression, which decreases the overall fitness of individuals endangering the species survival^29–32^. The threat of inbreeding depression and reduced genetic diversity in isolated wild populations has been well-documented, such as in wild house sparrows (*Passer domesticus*) (where inbreeding was associated with shorter telomeres)^33^, in Bengal tigers (*Panthera tigris tigris*) (where higher frequency of deleterious mutations were found in isolated populations)^34^, and even in the last woolly mammoth (*Mammuthus primigenius*) population^35^. Three out of four *P. sentosus* populations herein evaluated showed negative values of F_is_ which objectively indicate excess of heterozygotes than expected at random mating^36^, nevertheless this deviation can actually be due to genetic drift as these populations are small and retain low levels of genetic diversity^37^.

The genetic structure analyses indicated high levels of population structuring, which can be due to the impact of anthropogenic habitat modifications on the *P. sentosus* populations. High levels of population structuring have been previously observed in different reptile populations, including lizards and geckos of the family Phyllodactylidae^38,39^. Also, similar effects of urban fragmentation to those observed for *P. sentosus* have been reported in endemic geckos with restricted distribution such as the Mauritius lowland forest day gecko (*Phelsuma guimbeaui)* from the island of Mauritius, where its population was fragmented by urban expansion into small and isolated forest patches, resulting into populations with high genetic differentiation even in the absence of natural barriers^40^.

The historical gene flow results are largely consistent with this scenario, showing low relative migration rates between populations, particularly those at the extremes of the species distribution. This situation got established thousands of generations ago (see below), accelerating the population differentiation by genetic drift. Contemporary gene flow rates are lower than historical ones, showing that currently, the probability that an individual from a *huaca* could disperse to another is minimal, since the populations are isolated due to the presence of buildings, parks and roads acting as barriers for a species characterized by low vagility and dispersal capability^11^.

On the other hand, high levels of historical gene flow (0.99–1) were found between San Marcos and Parque de Las Leyendas populations, which is in agreement with population structure analyses where both populations clustered together. Interestingly, both localities were part of the pre-Hispanic Maranga archeological complex and were connected by unpaved areas until the 1950s, when the process of asphalting an avenue was completed and there was an increase in infrastructure in the area including the establishment of a zoo and two of the largest universities in Lima. Contemporary gene flow between these two localities is significantly lower (0.06–0.166), although higher than between most other localities, showing that connectivity between Parque de Las Leyendas and San Marcos stopped more recently. While during pre-hispanic settlement these huacas were connected by the *Qhapaq Ñan* (the Andean road system that expanded across six Andean countries), these ancient routes have been destroyed during Lima’s urban expansion, with the connection between Parque de Las Leyendas and San Marcos being one of the last remnants until 1950^41^.

In addition, when considering the four largest populations, the observed historical and contemporary migration values to and from Mateo Salado were lower, which is consistent with the high coefficients of genetic group membership (q) previously recorded for this locality (Fig. 3b,c), despite the proximity of the other localities (2.5 km). This marked population divergence exemplifies the power of genetic drift to generate divergent populations. This site was heavily impacted during decades by agriculture and other human activities including automobile repair shops, which could have had an even stronger isolating effect. This locality has since been redeemed in the last decade thanks to efforts to preserve the cultural-historic heritage.

The genetic population structuring, and low gene flow observed among populations could be explained by geographical reasons, since the species distribution is interrupted by the Rímac and Chillón rivers (Fig. 3). However, our results show a more structured scenario than the expected by these landscape features (Fig. 3). In the case of Chillón River, it seems as a natural barrier to gene flow since Tambo Inga (the only population recorded north Chillón River) has the lowest levels of migration compared to other localities. On the other hand, although higher migration values are expectedly observed between populations south of Rímac River than between these and those located north to this barrier, some of the latter populations showed higher migration rates to San Marcos and Parque de Las Leyendas (both south of the river) than to nearby populations. These results may suggest that the rivers may represent an unstable barrier, allowing gene flow in certain periods and acting as a barrier in others, depending on the fluctuation of the water flow. This type of river effect has already been proposed to explain the gene flow observed in the Uruguay marked gecko (*Homonota uruguayensis)* in the Brazilian and Uruguayan pampas^42^, in rodent populations^43^ and lizards^44^. Another explanation to the population structuring observed here could be an isolation by distance model; however, the Mantel test results indicate that geographic distance does not explain genetic differentiation among populations. Thus, the scenario of population structuring observed among the studied populations of *P. sentosus* cannot be a product of geographic distance alone. Therefore, the results obtained indicate that anthropogenic habitat modifications may represent an important barrier to the dispersal of this gecko.

**Figure 6:**
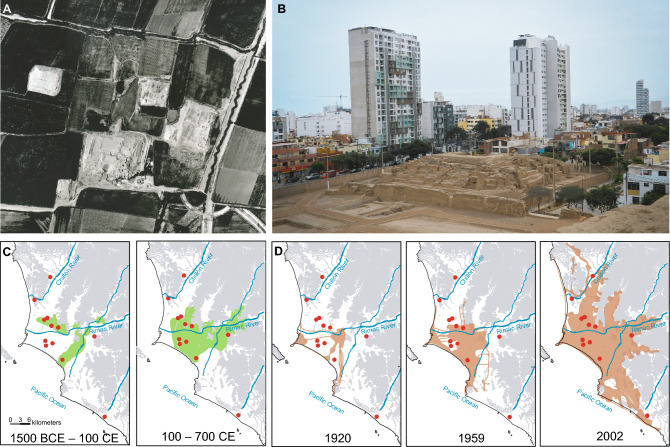
Habitat modification by human activity in the Rimac Valley, Lima (Peru). Landscape modification in the area has been done mainly by agricultural activities (**A**,**C**) and urbanization (**B**,**D**). (**A**) Photograph of the agricultural area surrounding the Mateo Salado *huaca* taken in 1944 by the National Aerophotographic Service of the Peruvian Air Force (FAP). (**B**) Current photograph of the Mateo Salado *huaca* surrounded by the Lima urban landscape. Photo by A. Arana. (**C**) The area around the Rímac River has been modified by pre-Incan human activity by the development of water canals for the spread of agricultural land since 1500 BCE (maps modified from ANA, 2016). Agricultural land established by pre-Incan cultures is depicted in green. (**D**) Further modification arose in the Rimac Valley by the establishment and growth of Lima city since 1920 (based on Sáez Giraldez et al., 2010). Modern urban environment is depicted in orange. Sampling localities of the Lima leaf-toed gecko are represented by red dots. Altitudinal relief is represented in gray lines (altitudes higher than 25 m). Maps created in ArcGIS 10.3. Free vector data from https://www.geogpsperu.com/2014/03/base-de-datos-peru-shapefile-shp-minam.html.

The habitat of *P. sentosus* has been modified constantly by the human occupancy during more than 5000 years. The city of Lima has been inhabited by several human cultures over time. The pre-Inca cultures developed aquifer systems that allowed them to sustain large areas of cultivated lands^41,45^. This water and soil management modified the landscape of Lima and, therefore, the habitats of local fauna and flora^46,47^. In the Late Archaic period (~ 4720 to ~ 3520 years ago), the domestication of plants and animals was already recorded in the Rímac valley, as well as the first irrigation canals built in the area^47^. Subsequently, in the Formative Period (~ 3520 to ~ 1920 years ago), large-scale hydraulic works and an expansion of intensive agriculture were built^47^ (Fig. 6). With the development of more recent urbanization, the modification of Lima's landscape has been more dramatic, including aspects such as buildings and roads^46,48^, isolating completely the remnant population of *P. sentosus* (Figs. 1, 6).

The genetic effect of these changes in the landscape can be observed in the demographic history of *P. sentosus* populations (Fig. 5). With the development of irrigation canals and agriculture in the area, this gecko*,* a specialist species adapted to arid areas, lost most of its habitat, but found refuge in the *huacas*, where population fragmentation, decrease in effective population size and loss of genetic diversity started. Bottleneck effects were observed in all populations analyzed during the last 5000 and 2000 years ago, except for Pucllana that experience a bottleneck between 10,000 and 5000 years ago. After the bottleneck all populations recovered and reached a stable size, showing a period of adaptations to the new environmental conditions. Based on global assessments^5,8^, it is expected that the new pressures brought from the urbanization and city growth (Fig. 6) will negatively affect the remnant populations. This process can be observed in Pucllana where a drop in the effective population size was estimated to occur about 200 years ago (Fig. 5). However, estimating with accuracy very recent demographic changes that are complex and could include several bottlenecks events is not possible with the method used in this study, which is more appropriate to detect demographic change at the scale of thousands of years^49^. Methods that can discern very recent demographic changes use linkage disequilibrium analysis and therefore require a reference genome^49–51^. This would be an important next step in the study of this endangered species.

Another factor that could have affected the landscape of the area, during this period is the El Niño Southern Oscillation (ENSO) event, which can produce flooding, variations in temperature and precipitation. However, climate simulations during the Holocene in South America show that in the central coast of Peru there was no major variation in precipitation and vegetation in the last 8000 years, unlike what happened in northern and southern Peru^52^, allowing an increase in human populations in the sub-continent^53^. Conversely, it has been estimated that floods occurred more frequently on the coast of Peru between 7000 to 8000 years ago^54,55^. The Pucllana population may have been affected by a flood caused by an ENSO event in years prior to the settlement of pre-Inca communities. Nonetheless, an event affecting only this locality is unlikely. Therefore, it is possible that the variation in the estimated dates of the bottleneck at Pucllana is due to bias effects related to recent migrations or admixture (see Fig. 2). This type of bias due to demographic processes has been detected in different types of demographic history analyses^56^.

The whole scenario for *P. sentosus* conservation does not seems to be favorable. Unlike the previous PVA^57^, in which no initial genetic diversity value was included, we included a starting initial genetic diversity value of 0.15 based on our genetic diversity results. PVA results showed a mean extinction time of less than 10 years, in contrast with the previous one, in which the mean extinction time was longer than 33 years (if extinct at all). The addition of an initial genetic diversity value causes the effects of this low diversity to be evident from the beginning and as the simulations progress^58^ these effects include inbreeding depression and the increased probability of lethal alleles occurrences^59^. This implies that, in a period of 10 years, if no changes are made in favor of its conservation and considering that there are no populations with more than 1000 individuals, all populations of *P. sentosus* would become extinct.

We must mention that these results are based on simulations performed by the Vortex program based on the information that is available about the natural history of *P. sentosus*^11,16,57^ and that they are not definitive results but are a prediction that may vary as more is known about this species and actions are taken to preserve it. The drastic decline in the following years is plausible because the species could enter an extinction vortex in which both a deterioration of population and genetic characteristics occur gradually^60,61^. In addition, the rate of population decline increases the closer the population gets to extinction^62^. These predictions represent a red flag for a species that need urgent conservation measures.

Nowadays, *P. sentosus* is only found in a few *huacas* and hills that maintain sandy substrate with rocks^11^. Most of these sites are legally protected for cultural reasons^63^, but still face several threats in practice such as illegal land appropriation and informal garbage dumps. Furthermore, protection measures may not necessarily align with the goal of conserving wildlife inhabiting the *huacas* and can either not address threats relevant to the Lima leaf-toed gecko (like the presence of invasive predators, e.g., cats and rats) or directly endanger them with the removal of the gecko’s retreat sites during archeological restoration.

Based on the results obtained, we recommend a management plan for the conservation of *P. sentosus* that incorporates the recovery of gene flow between populations through the translocation of individuals between localities. Management actions should be coordinated with national biodiversity authorities as with local archeological institutions, considering that most populations of *P. sentosus* live in archeological areas that are protected by the national government authorities. Also, our attention should be drawn to the populations in areas that do not have protection and that are at risk of disappearing. Another important future step should be producing a high-quality genome assembly, as this would provide more information about the genomic health of these populations (for example by analyzing the population frequency of runs of homozygosity and deleterious alleles to assess the effects of inbreeding), and about genomic signatures of local adaptation^64^. Finally, a management plan should be integrated with the ongoing efforts in captive breeding that have already produced protocols for artificial incubation for this species^65^ and future ex-situ conservation programs that include zoo-kept gecko populations to build insurance colonies for this endangered taxon^66^. This would benefit from implementing more monitoring, ecological and reproductive studies of *P. sentosus*. These studies are crucial for incorporating more precise information into population genetic analyses and population viability simulations, to gain a deeper understanding of the natural history of this species.

## Methods

### Sample collection and genomic DNA extraction

We collected 50 tissue samples from 12 *P. sentosus* localities in Lima, Peru (Fig. 1) under the research and sampling permit (SERFOR N° AUT-IFS-2018-40). We conducted opportunistic visual encounter surveys between 18:00 and 24:00 h by taking identification photos upon capture. We obtained more samples from localities where larger census population sizes were previously registered: San Marcos, Parque de Las Leyendas, Pucllana and Mateo Salado^16–18^. In contrast, much smaller census population sizes have been recorded in other localities (Pérez pers. commun.). We used sterilized surgical steel scissors to cut the tail tip up to 15 mm from the tip. In a few cases autotomized tail parts were collected. Geckos were all safely released after processing. Tissue samples were stored in 96% ethanol at − 18 °C prior to DNA extraction. We then extracted genomic DNA using an innuPREP DNA Mini Kit (Analytik Jena, Jena, Germany) and quantified the extracted DNA with NanoDrop Lite (Thermo Scientific, Waltham, USA).

### Library preparation

GBS libraries were generated via labor contract with University of Minnesota Genomics Center (https://genomics.umn.edu/services/gbs). Here we briefly describe the protocol, which is based on the one proposed by Elshire et al.^24^. A total of 100 ng of DNA was digested with 10 units of the restriction enzymes PstI-HF and MspI (New England Biolabs, Inc. MA, USA); and incubated at 37 °C for 2 h. DNA samples were ligated with 200 units of T4 ligase (New England Biolabs, Inc. MA, USA) and phase adaptors with -TGCA and CG overhangs at 22 °C for 1 h and heat killed. Ligated libraries were purified with SPRI beads and then amplified for 18 cycles with 2 × Taq Master Mix (New England Biolabs, Inc. MA, USA) to add the barcodes. GBS libraries were then purified, quantified using a NanoDrop™ Lite Spectrophotometer, and pooled. Fragments with the 300–744 bp size region were selected and diluted to 2 nM for sequencing on the Illumina NovaSeq 550 (Illumina, CA, USA) using 1 × 150 single-end reads.

### SNP genotyping and filtering

First, cutadapt^67^ and fastp^68^ were used to remove adapters. Then sequencing reads were processed using ipyrad v. 0.7.13^69^ with the following parameters: minimum depth for statistical base calling = 6; maximum heterozygous sites per locus = 0.5; clustering threshold = 0.85. We generated two data sets with ipyrad from 50 individuals from 12 localities, one dataset (i) with no missing data (loci shared by all individuals) and a second one (ii) with loci shared by at least 33 individuals (66% of the total number of individuals collected).

### Genetic diversity and inbreeding coefficient

Two measures of genetic diversity were applied at the population level using the population tool from Stacks^70^: (1) nucleotide diversity (π) defined as the mean number of differences per nucleotide site between two sequences from a pair of individuals randomly selected from a population^71,72^; and (2) expected heterozygosity (He). To facilitate a meaningful comparison with our results, we also calculated expected heterozygosity for four other *Phyllodactylus* species using data from a study conducted by Ramírez-Reyes et al. in 2020^73^. The inbreeding coefficient (F_IS_) indicating the deviation of the observed heterozygosity relative to the expected heterozygosity^36^, was also obtained using the Stacks tool set. F_is_ was calculated only for the four largest populations (largest census size^16–18^ and sample size of 10 individuals: Table 1).

### Population genetic structure

To analyze the genetic structure, a principal component analysis (PCA) was performed using the glPCA function of the R package adegenet^74^. We used the ipyrad-analysis toolkit for a t-SNE analysis, which is a big data visualization method developed by machine learning (Automatic Learning) within the field of artificial intelligence and has been proposed as a better method for visualizing SNPs than PCA^75,76^. Also, a discriminant analysis of principal components (DAPC) was conducted using the function dapc of the R package adegenet^74^, and the number of principal components that were retained for the DAPC was chosen with the function xvalDapc using the cross-validation method as recommended by Jombart and Collins^77^. Finally, a STRUCTURE^78^ analysis was performed using the ipyrad packages and the admixture model with a correlated allele frequency. Also, we used two different *mincov* options, filtering all missing data or maintaining only SNPs with 0.9 of sample coverage. The number of genetic groups (K) was determined by the largest ΔK^79^.

### Gene flow

We created a file in GENEPOP^80^ format from the vcf file using unlinked_snps_to_genepop from ipyrad tools (github.com/brunoasm/ipyrad_tools). We then examined the pairwise historical migration patterns of local populations using the divMigrate method^25^ implemented in the R package diveRsity^81^. This method estimates relative directional migration based on asymmetric distributions of allele frequencies^25^. Results are presented in relative migration rates with values between 0 and 1^25^. We made a modification in the code to allow the incorporation of populations that had a single individual as a sample (see Supplementary Material S6 online). GST was chosen among the migration measures offered by divMigrate^25^.

We used the Mantel test to assess if there was a correlation between geographical and genetic distances between populations, following the isolation by distance model (IBD)^82,83^ while using the R package hierfstat^84^ to obtain Nei's F_ST_, which was used as a measure of genetic distance. Geographic distances were obtained using the Geographic Distance Matrix Generator v. 1.2.3^85^. The Mantel test with the pairwise genetic distances (F_ST_/(1 − F_ST_)) and the pairwise geographic distances (log transformed) was performed using the Mantel function of the R package vegan^86^ with 10,000 permutations.

Contemporary gene flow was estimated using a modification of BayesAss 3.04^27^, called BA3-SNPs v. 3.0.4^26^, which accepts large SNP datasets and estimates migration rates as proportion of migrants using a Bayesian framework and Markov chain Monte Carlo (MCMC). We used BA3-SNP-autotune v 3.0.4^26^ to determine the optimal mixing parameters for our dataset for migration rates (deltaM = 0.9991), allele frequencies (deltaA = 0.7750), and inbreeding coefficients (deltaF = 0.0250). We ran BA3-SNPs for 100 million iterations, with a burn-in of 10 million. Chain convergence was assessed in Tracer v 1.7.1^87^.

### Demographic history

To infer the demographic history of the Lima leaf-toed gecko we first generated a folded SFS file with easySFS (https://github.com/isaacovercast/easySFS). We then used Stairway plot v. 2.1^88^ to estimate the historical effective population size changes of the largest gecko populations in Lima^16–18^: Huaca San Marcos, Parque de Las Leyendas, Mateo Salado and Huaca Pucllana. We did not analyze the gecko population as a whole, since high levels of population structure are considered factors that artificially modify the results^56^.

The parameters for the Stairway Plot analysis were as follows: 67% for the training sites; random breakpoints at 4, 9, 14, and 18; and one year was assumed as generation time (J Pérez, pers. commun.). The mutation rate was assumed to be 2.4 × 10^–9^, since this value is frequently used in studies on species of the order Squamata^89,90^.

### Population viability analysis

To carry out the Population Viability Analysis (PVA) we used Vortex (v. 10.2.13.0)^58^ which performs simulations, based on given conditions, for different populations. Five populations were simulated, each one with a different initial census population size (N0 = 50, 100, 200, 500, 1000) and a carrying capacity (K) that corresponds to 125% of the initial census population size. The maximum age was 4 years old. These conditions were chosen based on previous ecological population studies^16,18^ and previous PVAs^57^. Simulation parameters are summarized in Supplementary Table S7 (see online). These parameters were carefully selected based on an extensive review of the existing literature on *P. sentosus*^11,16,57^, and through in-depth consultations with experts actively engaged in the study of this species.

### Ethical statement

Field protocols for the capture, handling, release of Lima leaf-toed geckos, and tail tips sampling were approved by the competent authority: the National Forestry and Wildlife Service (SERFOR) of the Ministry of Agriculture and Irrigation of Peru (permit: SERFOR N° AUT-IFS-2018-40). All methods were performed in accordance with the relevant SERFOR guidelines and regulations, as well as in compliance with the IUCN Policy Statement on Research Involving Species at Risk of Extinction.

### Supplementary Information

Supplementary Information.

## Data Availability

Genotyping-By-Sequencing fastq data were deposited to GenBank under BioProject PRJNA939276. Other data generated and analyzed are presented in the figures or tables of Supplementary Material.
