# Supplementary material for: Population genomics reveals how 5 ka of human occupancy led the Lima leaf-toed gecko (Phyllodactylus sentosus) to the brink of extinction
Source: Sci Rep. 2023 Oct 27;13:18465. doi: 10.1038/s41598-023-45715-x (PMC10611785; doi:10.1038/s41598-023-45715-x)
Supplement: Supplementary file 1 — Supplementary Information. [file 41598_2023_45715_MOESM1_ESM.docx]

**Population genomics reveals how 5ka of human occupancy led the Lima leaf-toed gecko (*Phyllodactylus sentosus*) to the brink of extinction**

**Alejandra Arana^1^, Juan Esteves^1^, Rina Ramírez^1^, Pedro M. Galetti Jr. ^2^, José Pérez Z.^1^, Jorge L. Ramirez^1^**

1. Facultad de Ciencias Biológicas. Universidad Nacional Mayor de San Marcos, 375 German Amezaga, Lima, Peru

2. Departamento de Genética e Evolução, Universidade Federal de São Carlos, 13565-905, São Carlos, SP, Brazil. (ORCID: 0000-0001-5916-6126)

**Supplementary Information**


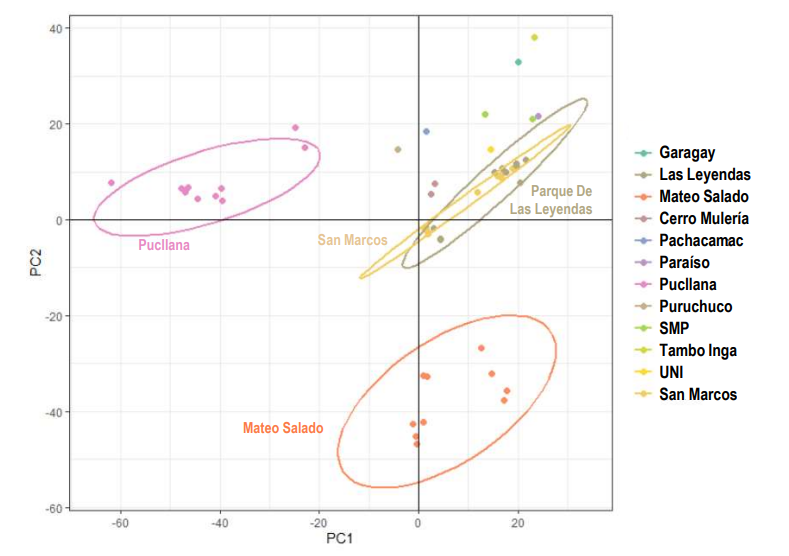


**Supplementary Figure S1.** PCA genetic structure analysis of Lima leaf-toed geckos *Phyllodactylus sentosus* populations. Each dot represents an individual assessed.


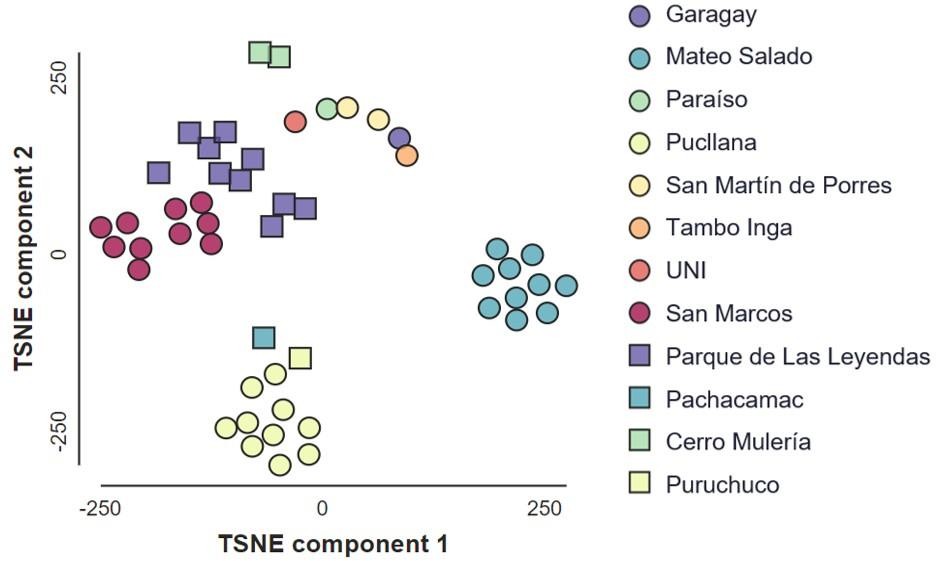


**Supplementary Figure S2.** t-SNE genetic structure analysis of Lima leaf-toed gecko *Phyllodactylus sentosus* populations. Each dot represents an individual assessed.

|  | **San Marcos** | **Paraíso** | **La Mulería** | **Parque de Las Leyendas** | **Pachacamac** | **Garagay** | **San Martín de Porres** | **Mateo Salado** | **Tambo Inga** | **UNI** | **Pucllana** | **Puruchuco** |
| --- | --- | --- | --- | --- | --- | --- | --- | --- | --- | --- | --- | --- |
| **San Marcos** | NA | 0.07180995 | 0.16962785 | 0.9908118 | 0.06619401 | 0.054731 | 0.1477185 | 0.29659323 | 0.04895928 | 0.07343412 | 0.31320588 | 0.06246973 |
| **Paraíso** | 0.17920988 | NA | 0.10337334 | 0.1688424 | 0.05534199 | 0.04653597 | 0.11552398 | 0.10472529 | 0.0470231 | 0.05788247 | 0.12371868 | 0.05085976 |
| **La Mulería** | 0.23214275 | 0.05843559 | NA | 0.2221563 | 0.05119283 | 0.0457775 | 0.09702144 | 0.13910816 | 0.04387598 | 0.05564169 | 0.16036126 | 0.04745608 |
| **Parque de Las Leyendas** | 1 | 0.0713074 | 0.16099598 | NA | 0.06780272 | 0.05472106 | 0.15478351 | 0.28171899 | 0.05030515 | 0.07218352 | 0.30722449 | 0.06474557 |
| **Pachacamac** | 0.13197923 | 0.05413233 | 0.07582191 | 0.1374559 | NA | 0.04717677 | 0.07597066 | 0.09369694 | 0.04663242 | 0.05402364 | 0.12563063 | 0.05536085 |
| **Garagay** | 0.11604251 | 0.05108526 | 0.07142516 | 0.1159761 | 0.05247767 | NA | 0.09632222 | 0.08591504 | 0.05329555 | 0.04979287 | 0.09424841 | 0.04959837 |
| **San Martín de Porres** | 0.2043341 | 0.07289459 | 0.11174874 | 0.212421 | 0.05448391 | 0.05613085 | NA | 0.12492613 | 0.05276221 | 0.05938988 | 0.14021536 | 0.05315173 |
| **Mateo Salado** | 0.39595623 | 0.05090252 | 0.10872337 | 0.3598385 | 0.0497321 | 0.04319855 | 0.09410459 | NA | 0.0381713 | 0.05366573 | 0.21701965 | 0.04980714 |
| **Tambo Inga** | 0.09825902 | 0.0506513 | 0.07064852 | 0.104434 | 0.05068081 | 0.0518923 | 0.08537927 | 0.07464433 | NA | 0.04769257 | 0.08850688 | 0.05014256 |
| **UNI** | 0.15816701 | 0.05759378 | 0.08996919 | 0.1503845 | 0.05445239 | 0.04564879 | 0.09734102 | 0.10624945 | 0.04471607 | NA | 0.12239474 | 0.05309603 |
| **Pucllana** | 0.40231985 | 0.06301611 | 0.12194774 | 0.3645238 | 0.06019111 | 0.05159704 | 0.11217007 | 0.21555512 | 0.04683035 | 0.06163965 | NA | 0.06701948 |
| **Puruchuco** | 0.12988365 | 0.05034181 | 0.06958314 | 0.1337196 | 0.05525922 | 0.04525356 | 0.07328111 | 0.09346591 | 0.04638164 | 0.05314029 | 0.12505767 | NA |

**Supplementary Table S3**. Matrix of relative mutation rate values (GST) among *Phyllodactylus sentosus* populations in Lima, calculated with divMigrate.

|  | **Mateo Salado** | **Pucllana** | **Leyendas** | **Garagay** | **Tambo Inga** | **Puruchuco** | **Paraiso** | **Pachacamac** | **UNI** | **Muleria** | **SMP** | **San Marcos** |
| --- | --- | --- | --- | --- | --- | --- | --- | --- | --- | --- | --- | --- |
| **Mateo Salado** | 0.8333 | 0.0152 | 0.0152 | 0.0258 | 0.0258 | 0.0255 | 0.0254 | 0.0258 | 0.0257 | 0.0235 | 0.0236 | 0.0154 |
| **Pucllana** | 0.0152 | 0.8181 | 0.015 | 0.0255 | 0.0258 | 0.0261 | 0.0256 | 0.0258 | 0.0256 | 0.0242 | 0.024 | 0.0152 |
| **Leyendas** | 0.0151 | 0.0305 | 0.7879 | 0.0508 | 0.0514 | 0.0514 | 0.0515 | 0.0513 | 0.0512 | 0.0238 | 0.071 | 0.166 |
| **Garagay** | 0.0153 | 0.0151 | 0.0153 | 0.6925 | 0.0256 | 0.0255 | 0.0262 | 0.0254 | 0.0259 | 0.0236 | 0.0241 | 0.0153 |
| **Tambo Inga** | 0.0152 | 0.0149 | 0.0151 | 0.0258 | 0.6925 | 0.0256 | 0.0255 | 0.0256 | 0.0258 | 0.0237 | 0.0237 | 0.0149 |
| **Puruchuco** | 0.0153 | 0.0152 | 0.0153 | 0.0257 | 0.0254 | 0.6925 | 0.0255 | 0.0259 | 0.0257 | 0.0236 | 0.0238 | 0.0151 |
| **Paraiso** | 0.0153 | 0.0153 | 0.0152 | 0.0256 | 0.0256 | 0.0256 | 0.6924 | 0.0254 | 0.0256 | 0.0241 | 0.024 | 0.0154 |
| **Pachacamac** | 0.0149 | 0.0152 | 0.0151 | 0.026 | 0.0256 | 0.0252 | 0.0254 | 0.6923 | 0.0259 | 0.0239 | 0.0241 | 0.0151 |
| **UNI** | 0.0149 | 0.0152 | 0.0152 | 0.0256 | 0.0252 | 0.0257 | 0.0257 | 0.0253 | 0.6921 | 0.0241 | 0.0236 | 0.0154 |
| **Muleria** | 0.0151 | 0.0153 | 0.0149 | 0.0253 | 0.0256 | 0.026 | 0.0256 | 0.0257 | 0.0255 | 0.6907 | 0.0239 | 0.0153 |
| **SMP** | 0.0151 | 0.0151 | 0.0152 | 0.0257 | 0.0257 | 0.0255 | 0.0257 | 0.026 | 0.0257 | 0.0235 | 0.6904 | 0.015 |
| **SanMarcos** | 0.0153 | 0.0151 | 0.0604 | 0.0258 | 0.0258 | 0.0255 | 0.0255 | 0.0255 | 0.0253 | 0.0712 | 0.0238 | 0.6819 |

**Supplementary Table S4**. Matrix of contemporary mutation rate values (BayeAss *m:* proportion of migrants) among *Phyllodactylus sentosus* populations in Lima, calculated with BA3-SNPs.


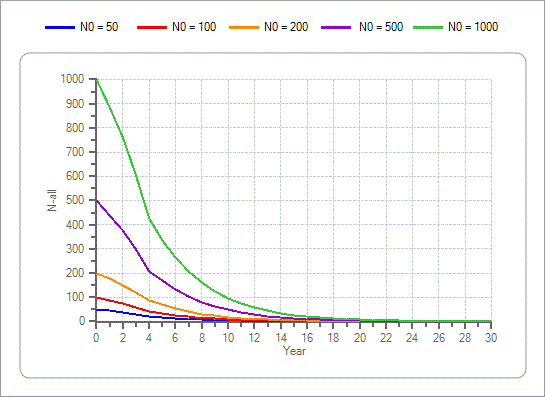


**Supplementary Figure S5.** Graph showing variation of population size of the five simulated populations of *Phyllodactylus sentosus*. N0: initial population, Nall: population size.

**Supplementary Material S6.** Modification of the code of the rpg.R file corresponding to the diveRsity package (the modification is highlighted in yellow).

############################################################################

# rpg: a new faster, memory efficient function for reading genepop files #

############################################################################

#' New readgenepop format

#'

#' Kevin Keenan 2014

rgp <- function(infile){

fastScan <- function(fname) {

s <- file.info(fname)$size

buf <- readChar(fname, s, useBytes = TRUE)

# replace Mac encoded line endings

if(length(grep("\r", buf)) != 0L){

buf <- gsub("\r", "\n", buf)

buf <- gsub("\n\n", "\n", buf)

}

return(strsplit(buf, "\n", fixed = TRUE, useBytes = TRUE)[[1]])

}

if(is.list(infile)){

infile <- as.matrix(infile)

dat <- apply(infile, 1, function(x){

x <- x[!is.na(x)]

return(paste(x, collapse = "\t"))

})

#dat <- c(paste(colnames(infile), collapse = "\t"), dat)

} else {

dat <- fastScan(infile)

# strip whitespace from the beginning an end of lines

dat <- sapply(dat, function(x){

sub("^\\s+", "", x)

})

dat <- sapply(dat, function(x){

return(sub("\\s+$", "", x))

})

names(dat) <- NULL

}

popLoc <- grep("^([[:space:]]*)pop([[:space:]]*)$", tolower(dat))

if(popLoc[1] == 3){

if(length(strsplit(dat[4], split = "\\s+")[[1]][-1]) > 1){

locs <- strsplit(dat[2], split = "\\s+")[[1]]

if(length(locs) == 1){

locs <- strsplit(dat[2], split = ",")[[1]]

}

locs <- as.character(sapply(locs, function(x){

x <- strsplit(x, split = "")[[1]]

if(is.element(",", x)){

x <- x[-(which(x == ","))]

}

return(paste(x, collapse = ""))

}))

dat <- c(dat[1], locs, dat[-(1:2)])

}

} else {

locs <- as.character(dat[2:(popLoc[1]-1)])

}

# strip whitespace from locus names

locs <- as.character(sapply(locs, function(x){

return(strsplit(x, split = "\\s+")[[1]][1])

}))

# npops

popLoc <- grep("^([[:space:]]*)pop([[:space:]]*)$", tolower(dat))

npops <- length(popLoc)

no_col <- length(locs)+1

nloci <- length(locs)

# get genotypes

strt <- popLoc + 1

ends <- c(popLoc[-1] - 1, length(dat))

genoRet <- function(strt, ends, x){

out <- strsplit(x[strt:ends], split = "\\s+")

x <- do.call("rbind", c(out, deparse.level = 0))

if(round(mean(nchar(x[,2]))) == 1L){

x[,1] <- paste(x[,1], x[,2], sep = "")

x <- x[,(-2)]

}

x[x == "-9"] <- NA

x[x == "0000"] <- NA

x[x == "000000"] <- NA

# output

####modified#####

if(nrow(x)==1){

y <- x[,(-1)]

y <- strsplit(y, split = "\\s+")

y <- do.call("cbind", c(y, deparse.level = 0))

} else {

y <- x[,(-1)]

}

list(ls = y,

nms = as.vector(x[,1]))

#list(ls = x[,(-1)],

#nms = as.vector(x[,1]))

####modified#####

list(ls = x[,(-1)],

nms = as.vector(x[,1]))

}

genos <- mapply(genoRet, strt = strt, ends = ends,

MoreArgs = list(x = dat), SIMPLIFY = FALSE)

indNames <- lapply(genos, "[[", 2)

#indNames <- do.call("c", indNames)

genos <- lapply(genos, "[[", 1)

# detect genepop format

# check for loci with all missing data before calculating gp

badLoc <- apply(genos[[1]], 2, function(x){

sum(is.na(x)) == length(x)

})

badLoc <- which(!badLoc)

gp <- round(mean(nchar(na.omit(genos[[badLoc[1]]][,1]))/2))

# convert genotypes to arrays

genos <- lapply(genos, function(x){

al1 <- substr(x, 1, gp)

al2 <- substr(x, (gp+1), (gp*2))

out <- array(NA, dim = c(nrow(x), ncol(x), 2))

out[,,1] <- al1

out[,,2] <- al2

return(out)

})

# calculate allele frequencies, obs alleles, popSizes

# define function

statFun <- function(x, cl = NULL){

# if(!is.null(cl)){

# tab <- parLapply(cl, 1:dim(x)[2], function(i){return(table(x[,i,]))})

# } else {

#tab <- lapply(1:dim(x)[2], function(i){return(table(x[,i,]))})

#}

popSizes <- apply(x, 2, function(y){

length(na.omit(y[,1])) * 2

})

af <- lapply(1:dim(x)[2], function(i){

y <- as.vector(na.omit(x[,i,]))

nms <- unique(y)[order(unique(y))]

ot <- myTab(y)

names(ot) <- nms

return(ot)

})

popSizes <- popSizes/2

list(af = af, ps = popSizes)

}

# rearrange data by loci

check <- function(args, gp){

#args <- list(...)

npops <- length(args)

pad <- paste("%0", gp, "g", sep = "")

rnames <- sprintf(pad,

unique(sort(as.numeric(unlist(lapply(args,

names))))))

out <- matrix(0, nrow = length(rnames), ncol = npops)

rownames(out) <- as.character(rnames)

for(i in 1:npops){

out[match(names(args[[i]]), rownames(out)),i] <- as.numeric(args[[i]])

}

return(out)

}

# calculate stats

obsAllSize <- lapply(genos, statFun)

# get individual stats

af <- lapply(obsAllSize, function(x){

out <- x$af

x$af <- NULL

return(out)

})

#obs <- lapply(obsAllSize, function(x){

# return(x$obs)

#})

ps <- lapply(obsAllSize, function(x){

out <- x$ps

x$ps <- NULL

return(out)

})

af <- lapply(1:(nloci), function(i){

return(lapply(af, "[[", i))

})

#obs <- lapply(1:(nloci), function(i){

# return(lapply(obs, "[[", i))

#})

ps <- lapply(1:(nloci), function(i){

return(sapply(ps, "[", i))

})

af <- lapply(af, check, gp = gp)

# names(af) <- locs

#obs <- lapply(obs, check)

gc()

list(af = af, genos = genos, ps = ps, gp = gp,

indnms = indNames, locs = locs)

}

############################################################################

# end rpg #

############################################################################

**Supplementary Table S7.** Number of individuals per population during 30 years in the five simulated populations in Vortex populations simulated in Vortex.

| **Year** | **Populations** | | | | |
| --- | --- | --- | --- | --- | --- |
| **0** | 50.00 | 100.00 | 200.00 | 500.00 | 1000.00 |
| **1** | 44.60 | 87.33 | 178.60 | 439.10 | 882.79 |
| **2** | 37.70 | 74.28 | 150.50 | 375.20 | 759.57 |
| **3** | 30.10 | 58.46 | 120.00 | 296.90 | 602.55 |
| **4** | 21.10 | 41.02 | 85.11 | 208.20 | 426.71 |
| **5** | 16.70 | 32.64 | 68.47 | 169.70 | 336.33 |
| **6** | 12.80 | 25.54 | 53.43 | 133.40 | 265.25 |
| **7** | 9.27 | 18.72 | 40.56 | 102.00 | 207.90 |
| **8** | 6.76 | 13.74 | 30.23 | 78.43 | 161.61 |
| **9** | 5.03 | 10.44 | 23.96 | 61.73 | 123.87 |
| **10** | 3.47 | 7.37 | 17.50 | 48.16 | 95.81 |
| **11** | 2.40 | 5.50 | 12.98 | 37.62 | 73.79 |
| **12** | 1.67 | 4.10 | 9.41 | 29.31 | 57.32 |
| **13** | 1.12 | 2.98 | 6.71 | 22.11 | 44.75 |
| **14** | 0.76 | 2.13 | 5.21 | 16.18 | 34.47 |
| **15** | 0.47 | 1.54 | 3.81 | 12.30 | 26.24 |
| **16** | 0.32 | 1.03 | 2.84 | 9.04 | 19.80 |
| **17** | 0.20 | 0.72 | 2.06 | 6.82 | 14.70 |
| **18** | 0.11 | 0.48 | 1.51 | 5.05 | 11.55 |
| **19** | 0.06 | 0.32 | 1.04 | 3.89 | 8.59 |
| **20** | 0.04 | 0.19 | 0.71 | 2.79 | 6.61 |
| **21** | 0.02 | 0.10 | 0.49 | 1.90 | 4.57 |
| **22** | 0.02 | 0.06 | 0.32 | 1.37 | 3.33 |
| **23** | 0.01 | 0.05 | 0.22 | 1.06 | 2.40 |
| **24** | 0.01 | 0.03 | 0.15 | 0.75 | 1.92 |
| **25** | 0.00 | 0.01 | 0.10 | 0.57 | 1.42 |
| **26** | 0.00 | 0.01 | 0.07 | 0.43 | 1.11 |
| **27** | 0.00 | 0.00 | 0.05 | 0.29 | 0.79 |
| **28** | 0.00 | 0.00 | 0.03 | 0.19 | 0.58 |
| **29** | 0.00 | 0.00 | 0.02 | 0.10 | 0.43 |
| **30** | 0.00 | 0.00 | 0.01 | 0.06 | 0.30 |
